# Supplementary figures and images for: Immunization with SARS-CoV-2 Nucleocapsid protein triggers a pulmonary immune response in rats
Source: PLoS One. 2022 May 24;17(5):e0268434. doi: 10.1371/journal.pone.0268434 (PMC9129034; doi:10.1371/journal.pone.0268434)

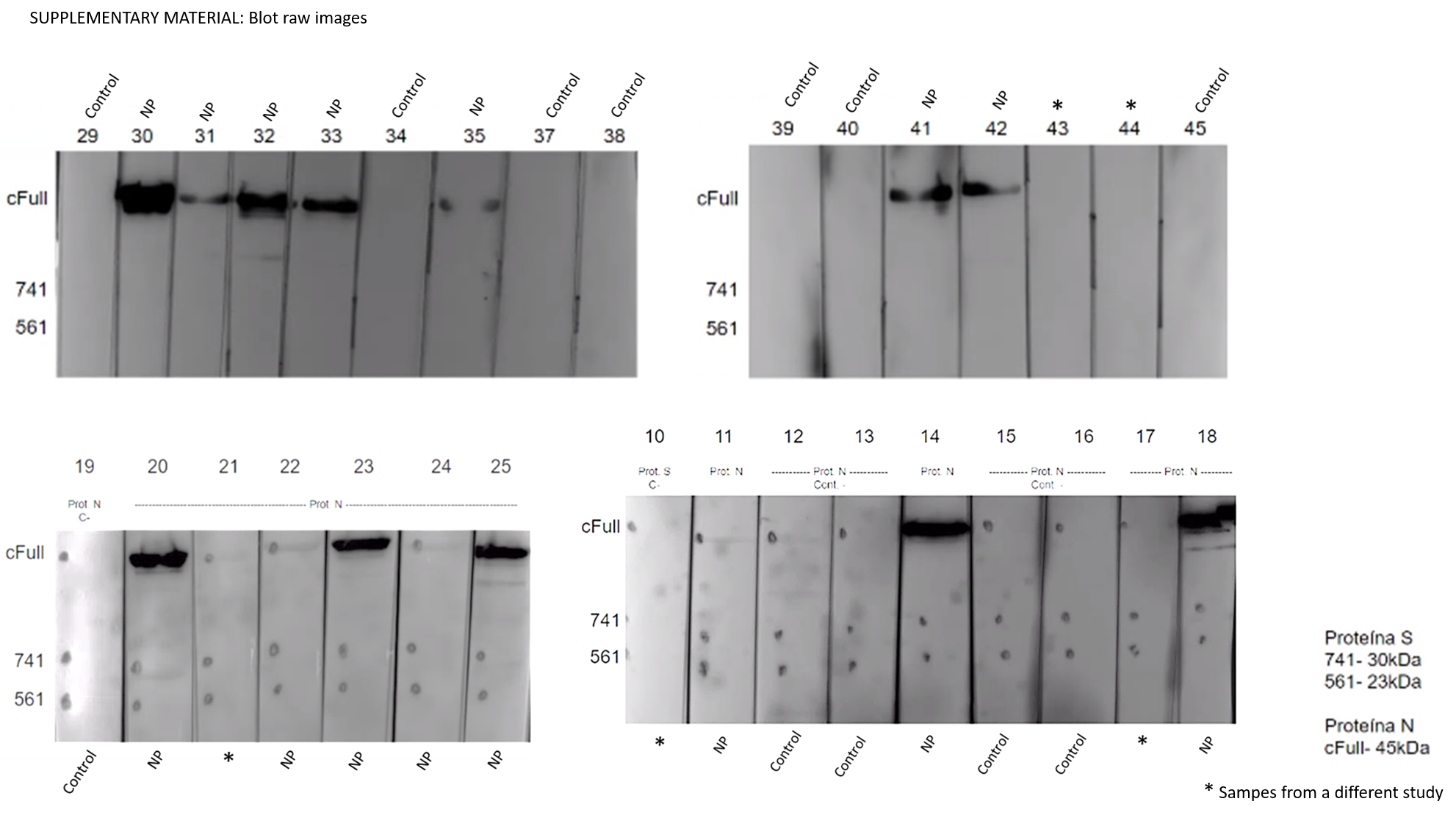

Supplement: S1 Raw images — (TIF) [file pone.0268434.s001.tif]
